# Selective Reduction of a Heterotopic Cesarean Scar Pregnancy Complicated by Septic Abortion

**DOI:** 10.1155/2018/6478589

**Published:** 2018-10-21

**Authors:** Joan Tymon-Rosario, Meleen Chuang

**Affiliations:** ^1^Montefiore Medical Center, Department of Obstetrics & Gynecology and Women's Health, Bronx, NY, USA; ^2^Albert Einstein College of Medicine, Bronx, NY, USA

## Abstract

**Background:**

Heterotopic pregnancy involving the implantation of an ectopic pregnancy into a prior cesarean scar with a concurrent intrauterine pregnancy is a rare and potentially life-threatening condition with minimal information in the literature to guide treatment and management options.

**Case:**

A 40-year-old G5P3103 at 12 weeks and 3 days with a history of two cesarean deliveries was diagnosed with a live heterotopic pregnancy containing a cesarean scar ectopic and an intrauterine pregnancy. After selective reduction of the cesarean scar gestation with potassium chloride (KCl), the patient presented ten days later to the emergency department with septic abortion and sepsis. The patient underwent bilateral uterine artery embolization followed by ultrasound guided uterine evacuation with dilation and curettage, which was complicated by intraoperative hemorrhage and persistent bacteremia. The patient had resolution of her bacteremia after total abdominal hysterectomy.

**Conclusion:**

Conservative management of uterine infection resulting from selective reduction of a heterotopic pregnancy cesarean scar pregnancy may be considered; however, severe septicemia and persistent bacteremia may necessitate definitive surgical management.

## 1. Introduction

A heterotopic pregnancy refers to the presence of simultaneous pregnancies at two different implantation sites, with the majority of ectopic pregnancies located in the fallopian tube. The literature to date involves discussion of the management of the more “traditional” heterotopic pregnancies with successful results. There is growing number of case reports of cesarean scar pregnancy with concurrent intrauterine pregnancies with variable clinical outcomes. Given the rise in cesarean sections and usage of assisted reproductive techniques over the past decades, this clinical conundrum will continue to increase in incidence. The rarity of a heterotopic pregnancy with one gestation being cesarean scar pregnancy (CSP) inherently limits evidence based recommendations on management options as well as all the potential adverse outcomes of various treatment options. We report a unique clinical circumstance of the implantation of an ectopic pregnancy into a prior cesarean scar with a concurrent intrauterine pregnancy and the subsequent complications that arose after selective reduction that ultimately necessitated hysterectomy.

## 2. Case

**Figure 1:**
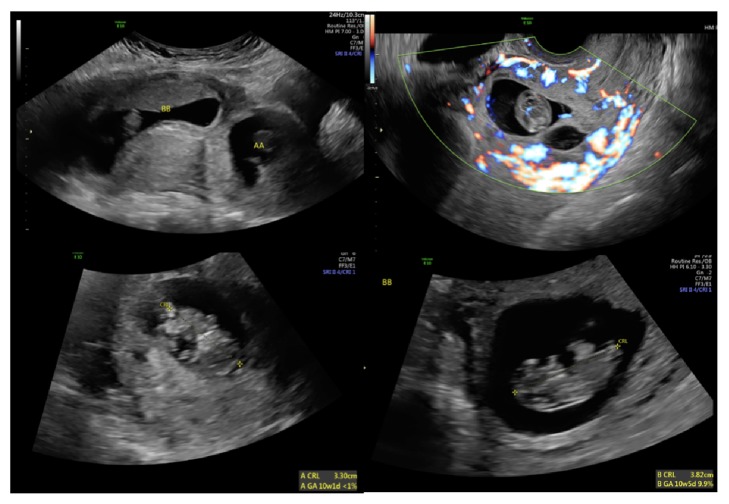
Sonographic imaged before selective reduction of cesarean scar pregnancy with potassium chloride (KCl injection). Upper left demonstrates cesarean scar pregnancy (AA) and intrauterine pregnancy (BB). Upper right demonstrates that cesarean scar pregnancy in close proximity to serosal edge of the uterus. Lower right and left demonstrate live pregnancy of Twin A (cesarean scar pregnancy) and Twin B (intrauterine pregnancy).

A 40-year-old G5P3103 at 12 weeks and 3 days with a history of two prior cesarean sections and known heterotopic pregnancy consisting of cesarean scar pregnancy and intrauterine pregnancy (Figure 1) presented ten days after successful selective reduction of cesarean scar pregnancy with potassium chloride (KCl) injection in the ultrasonography unit.

The patient reported two days of fevers prior to her presentation with new onset vaginal bleeding. After her initial visit after selective reduction, she was treated with Nitrofurantoin for a urinary tract infection at urgent care. She presented two days after urgent care to our emergency department (ED) with pain and ultrasonographic evaluation that demonstrated no fetal heartbeat and discharge home for follow-up with her provider for management options. Three days after ED visit, she represented with new onset fevers, chills, back pain, and scant vaginal bleeding. She denied any significant past medical history and her previous surgical procedure included gastric bypass surgery, two cesarean sections, and an endometrial ablation for heavy menses.

**Figure 2:**
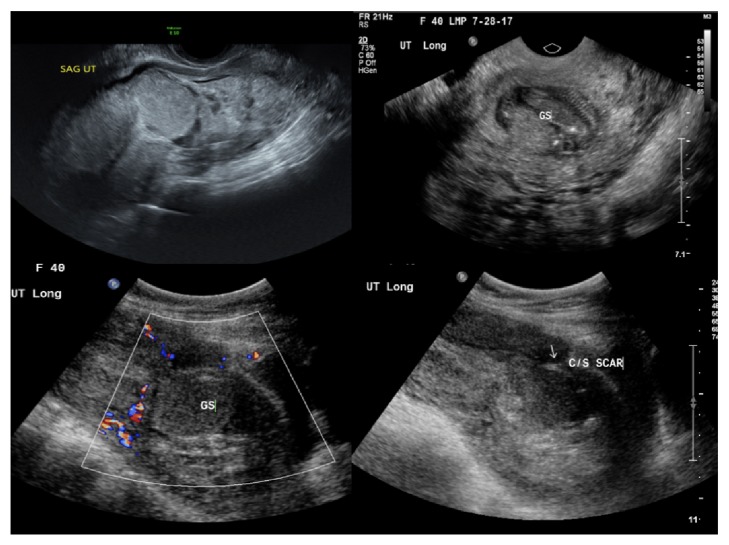
Sonographic findings when patient presented to the emergency room initially with the heterotopic gestation with a C-section scar ectopic containing fetal parts without discernible heart rate and an abnormal appearing intrauterine gestational sac in the lower uterine segment containing only low-level echoes.

On physical examination, the patient was febrile, tachycardic, hypotensive, and being in septic shock. She had scant dark blood in the vaginal vault, a 16 week size uterus with fundal tenderness. Ultrasound confirmed presence of no fetal cardiac activities and presence of high vascular flow to the myometrium surrounding the cesarean scar pregnancy (Figure 2).

**Figure 3:**
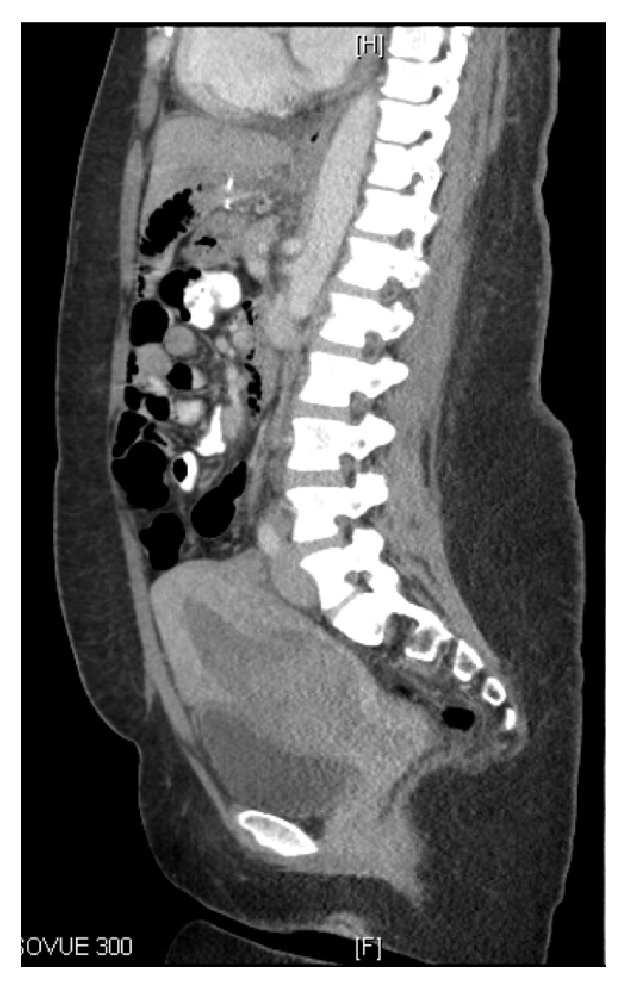
CT A/P with IV contrast with sagittal view demonstrating distention of the lower uterine segment of endometrial cavity.

The patient was counseled on septic abortion and she underwent a complete infectious disease workup, including blood cultures, urine cultures, and chest x-ray, and was started on broad-spectrum antibiotics (ampicillin, gentamicin, and clindamycin). Initial urine culture demonstrated no growth and initial blood culture grew* Enterococcus faecalis*. The patient desired uterine preservation and underwent bilateral uterine artery embolization using absorbable gelfoam and scheduled dilation and curettage under sonographic guidance the following day. The procedure was complicated with intraoperative hemorrhage of 1000cc that resolved with uterotonic medications and blood transfusion. Final pathology was consistent with products of conception. Hospitalized postoperatively, she continued to have daily low- grade fevers while on antibiotics with persistent daily positive blood cultures with* Enterococcus faecalis.* Antibiotic regimen was changed based upon the sensitivities to ampicillin and ceftriaxone, a normal echocardiogram ruled out endocarditis, and repeat transvaginal ultrasound demonstrated a large amount of heterogeneous avascular material in the lower uterine segment. The postoperative serum beta-hCG was 896 IU/L. CT scan of the abdomen and pelvis demonstrated a large high-density material within the endometrial cavity of lower uterine segment (Figure 3).

**Figure 4:**
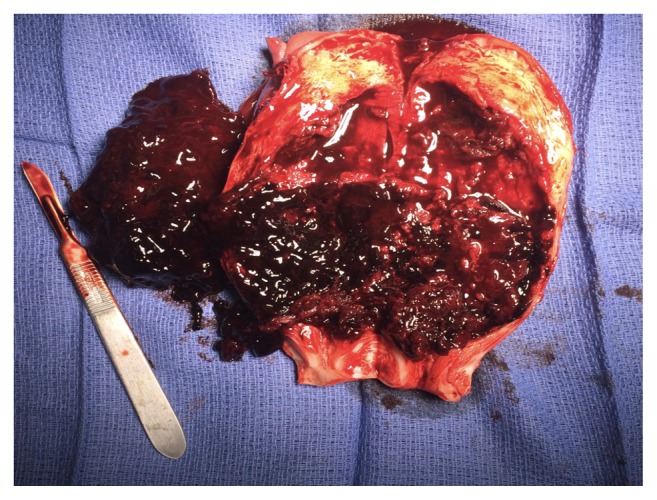
Bivalved uterus specimen shows anterior placenta adherent to lower uterine segment.

With concerns for persistent bacteremia, failed antibiotic therapy, and retained materials within the uterine cavity, the patient underwent a total abdominal hysterectomy and bilateral salpingectomy. The specimen was bivalved to show the entire uterine cavity with large amounts of blood clots, adherent placental tissue as well as a very thin anterior uterine segment (Figure 4).

Final pathology demonstrated products of conception with associated chronic and acute inflammation. Uterine culture obtained at hysterectomy demonstrated growth of* Enterococcus faecalis*, confirming the uterus as source of bacteremia. The patient had resolution of her fevers and negative blood cultures after the hysterectomy. Intravenous antibiotics were discontinued on postoperative day four and transitioned to a two-week course of Augmentin. She was discharged home with a two-week office follow-up. At her postoperative check and six-week visit she recovered fully.

## 3. Discussion

Selective reduction of heterotopic cesarean scar pregnancies via abortifacient injection, embryo aspiration, or both has been shown in the literature to be effective with multiple case reports citing success [1–5]. There are three case reports describing the successful management of a heterotopic CSP with selective reduction via potassium chloride injection of the cesarean scar pregnancy with preservation of the intrauterine gestation [1, 4, 6]. Hsieh et al. presented a successful case of embryo aspiration under sonographic guidance for selective embryo reduction in the setting of a heterotopic cesarean scar pregnancy with a combined intrauterine pregnancy [5].

The successful selective reduction of a heterotopic cesarean scar pregnancy via ultrasound guided potassium chloride (KCl) injection has even been reported in the second trimester [6]. Our case presents complications that arose from conservative management of sepsis as a result of selective reduction of a heterotopic cesarean scar pregnancy with sepsis and postprocedure bacteremia. The usual complication expected from conservative management of cesarean scar pregnancies is hemorrhage requiring blood transfusion and hysterectomy secondary to a morbidly adherent placenta [2, 7]. Our patient chose selective reduction after being provided with the evidence of previous successful selective reductions as well as the associated risks such as miscarriage, infection, and heavy bleeding possibly necessity embolization or hysterectomy.

Other conservative options of CSP's besides medical management have previously been reported in the literature. These options include methotrexate followed by uterine curettage, wedge resection via laparotomy or laparoscopy, uterine artery embolization followed by subsequent dilation, and curettage or systemic methotrexate with hysteroscopic or resection [8–11].

While Yu et al. have previously demonstrated the successful selective reduction of a heterotopic cesarean scar pregnancy in the second trimester; our case report is unique in that it illustrates the subsequent development of septic abortion and attempted conservative management of such a complication. The persistent bacteremia from the retained products of conceptions after the curettage is a lesser known complication that has not been discussed in the literature. This case report demonstrates that septic abortion, sepsis, and bacteremia resulting from selective reduction of a heterotopic cesarean scar pregnancy are an important complication to consider. This potentially life-threatening complication requires diligent management with active patient counseling and reassessment of the patient's clinical status and if medical management ultimately fails hysterectomy is warranted.

## Additional Points

*Teaching Points. *(1) Selective reduction may be an option to treat cesarean scar ectopic pregnancy for women desiring conservative management. (2) Septic abortion resulting from selective reduction of a heterotopic cesarean scar pregnancy is an important complication to consider. (3) Persistent bacteremia from retained products after surgical curettage of cesarean scar ectopic may necessitate definitive surgical management with hysterectomy.
